# The Effect of Diluted Deoxycholic Acid on Arm Fat Reduction: Evaluation of Its Potential in Minimally Invasive Fat Loss Treatment

**DOI:** 10.1111/jocd.70088

**Published:** 2025-02-27

**Authors:** Ji Yeon Hong, Yoon Hwan Lee, Seung Hoon Yeoum, Choon Shik Youn, Kui Young Park

**Affiliations:** ^1^ Department of Dermatology Chung‐Ang University Hospital, Chung‐Ang University College of Medicine Seoul Republic of Korea; ^2^ Songpajeil Orthopedic Clinic Seoul Republic of Korea; ^3^ Yemiwon Dermatologic Clinic Seoul Republic of Korea

**Keywords:** arm fat reduction, body contouring, deoxycholic acid, minimally invasive

## Abstract

**Background:**

Deoxycholic acid (DCA) at a concentration of 10 mg/mL is commonly used for localized fat reduction, but its application in larger areas like the upper arm can lead to higher costs and discomfort. Diluting DCA may provide a cost‐effective solution with reduced pain while still maintaining efficacy.

**Objective:**

This case series aims to evaluate the efficacy, safety, and overall cost‐effectiveness of diluted DCA injections at concentrations of 5 mg/mL and 2.5 mg/mL for upper arm fat reduction.

**Methods:**

Four healthy adult females received subcutaneous injections of either 5 mg/mL or 2.5 mg/mL DCA, administered three times at four‐week intervals. Arm circumference and subcutaneous fat thickness were measured at baseline and at 4, 8, 12, and 20 weeks using tape measures and ultrasonography. Pain levels and patient satisfaction were also assessed to gauge the overall balance between treatment efficacy, side effects, and costs.

**Results:**

Both 5 mg/mL and 2.5 mg/mL concentrations led to significant reductions in subcutaneous fat thickness, with the 5 mg/mL group showing slightly greater reductions. However, changes in arm circumference were minimal across both groups. Pain levels were higher in the 5 mg/mL group, while the 2.5 mg/mL group experienced less discomfort. Importantly, both concentrations demonstrated a balance between efficacy and treatment cost, with the diluted solutions providing a less invasive alternative to the standard 10 mg/mL concentration.

**Conclusion:**

This case series represents diluted DCA injections, both at 5 mg/mL and 2.5 mg/mL, offering viable minimally invasive options for upper arm fat reduction. While the 5 mg/mL concentration shows slightly greater efficacy, the 2.5 mg/mL option may offer a more comfortable treatment experience. The choice of concentration can be tailored to patient priorities, balancing fat reduction, pain tolerance, and cost considerations.

## 1 Introduction

Weight fluctuations and aging can cause the development of excess fat and loose skin in the upper arms, which is a significant concern for individuals pursuing a slim body contour. The desire for well‐contoured upper arms has become increasingly prevalent, driven by societal beauty standards and the popularity of sleeveless fashion. This trend is evidenced by a significant rise in upper arm lift procedures, with nearly 20,000 performed in 2019—a 20% increase from 2015 [1]. With the growing demand for noninvasive body contouring procedures, various techniques such as cryolipolysis, high‐intensity focused ultrasound (HIFU), radiofrequency, and injection lipolysis have been developed to target localized fat deposits. Cryolipolysis has been shown to effectively reduce arm fat through controlled cooling‐induced adipocyte apoptosis, though its efficacy depends on precise applicator placement [2, 3]. HIFU, another widely used technique, achieves fat reduction by inducing thermal coagulation of adipocytes, with studies suggesting that its effects can be enhanced when combined with electrical stimulation to accelerate lipid metabolism [4]. These noninvasive methods offer alternatives to surgical options like liposuction but may require multiple sessions to achieve optimal results.

Deoxycholic acid (DCA) is a secondary bile acid converted by gut microorganisms and involved in the emulsification and solubilization of dietary fats. When injected into the subcutaneous fat, DCA disrupts adipocyte cell membranes, leading to cell lysis. Subsequently, macrophages clear the remaining cellular and lipid debris, followed by fibroblast‐mediated thickening of fibrous septa, indicative of neocollagenesis. Currently, DCA at a concentration of 10 mg/mL is FDA‐approved as a lipolytic agent for submental fat reduction [5]. Recent studies have demonstrated the efficacy of DCA injections for localized fat reduction in various body areas. Its application has shown promising results in noninvasive body contouring, including bra‐line lipolysis, suggesting its potential for targeted adipolysis in different regions [6]. Judging from the cases of DCA injections for fat reduction in submental and other areas, it is also expected to be effective in reducing fat in the upper arm. However, since the upper arm area is larger than the submental area, a larger quantity of the drug is needed, leading to a higher treatment cost [7]. To the best of our knowledge, there are no studies assessing upper arm fat reduction using different DCA concentrations.

In this case series, we aim to assess the efficacy, safety, and effective concentration for reducing upper arm fat through diluted DCA injections in adult patients seeking cosmetic fat reduction in the upper arm.

## 2 Materials and Methods

### 2.1 The Study Design

Eligible subjects were Korean female healthy adults aged 19–65 years who desired to reduce their upper arm fat, had a body mass index (BMI) < 35, and self‐rated their upper arm satisfaction score ranging from 1 to 3 on the subject satisfaction scale. Exclusion criteria included patients with a previous fat reduction procedure (e.g., liposuction, surgery, or lipolytic agent injection), a trauma history near the treatment area, and a history of radiofrequency, laser, chemical peel, or dermal filler application to the upper arm area within the past year, as well as botulinum toxin injection to the upper arm or deltoid area within the last 24 weeks. During the study, subjects were instructed to avoid any medications or procedures that might influence the outcome of the study.

### 2.2 Intervention

Each vial containing 20 mg of DCA (V‐OLET, HanAll Biopharma, Seoul, Korea) was diluted to optimize both efficacy and cost‐effectiveness for broader applications beyond submental fat reduction. To achieve this, the solution was diluted based on practical clinical experience at a ratio of 1:1 or 1:3 with sterile saline, resulting in concentrations of 5 mg/mL and 2.5 mg/mL, respectively. Patients were randomized into two groups and received diluted DCA subcutaneous injections into both arms three times, each 4 weeks apart. EMLA 5% cream (lidocaine 25 mg/g, prilocaine 25 mg/g) was applied 30 min before the injection, followed by cleansing the skin with ethanol. After marking 25 points evenly spaced 1 cm apart on the back of the upper arm, 4 cm above and below the center of the acromion and olecranon, each participant received diluted V‐OLET (5 mg/mL or 2.5 mg/mL) in the subcutaneous fat using a 31‐gauge insulin syringe at 0.2 mL per point, for a total of 10 mL for both arms.

### 2.3 Assessment

Participants visits occurred at baseline, 4, 8, 12, and 20 weeks. At each visit, photographs were taken using a standardized photographic setup with the arm raised horizontally in a standing position. Participants were positioned using a cradle to ensure that the shoulder and wrist were at the same level. Additionally, treatment efficacy was objectively measured using a tape measure and ultrasonography at each visit. The midpoint of the acromion and olecranon was measured using a tape measure to obtain arm circumference. With the patient in the prone position, the ultrasound probe was positioned longitudinally in the most prominent area above an imaginary line connecting the end of the shoulder to the elbow. The thickness of the subcutaneous fat was determined as the average of the four measurements at the boundary line, dividing the ultrasound image screen into five equal parts. Pain intensity was also evaluated by the participants using a visual analog scale (VAS), ranging from 0 (no pain) to 10 (extreme pain) during treatment. Participant satisfaction was assessed immediately following the treatment and again after 4 weeks. Satisfaction levels were classified as 1 for completely dissatisfied, 2 for dissatisfied, 3 for somewhat dissatisfied, 4 for neither satisfied nor dissatisfied, 5 for somewhat satisfied, 6 for satisfied, and 7 for completely satisfied. Participants were requested to report any discomfort or adverse effects, including foreign body/burning sensation, erythema, or skin necrosis, during and after the procedure. Regular physical examinations were conducted as a part of safety monitoring.

## 3 Results

**FIGURE 1:**
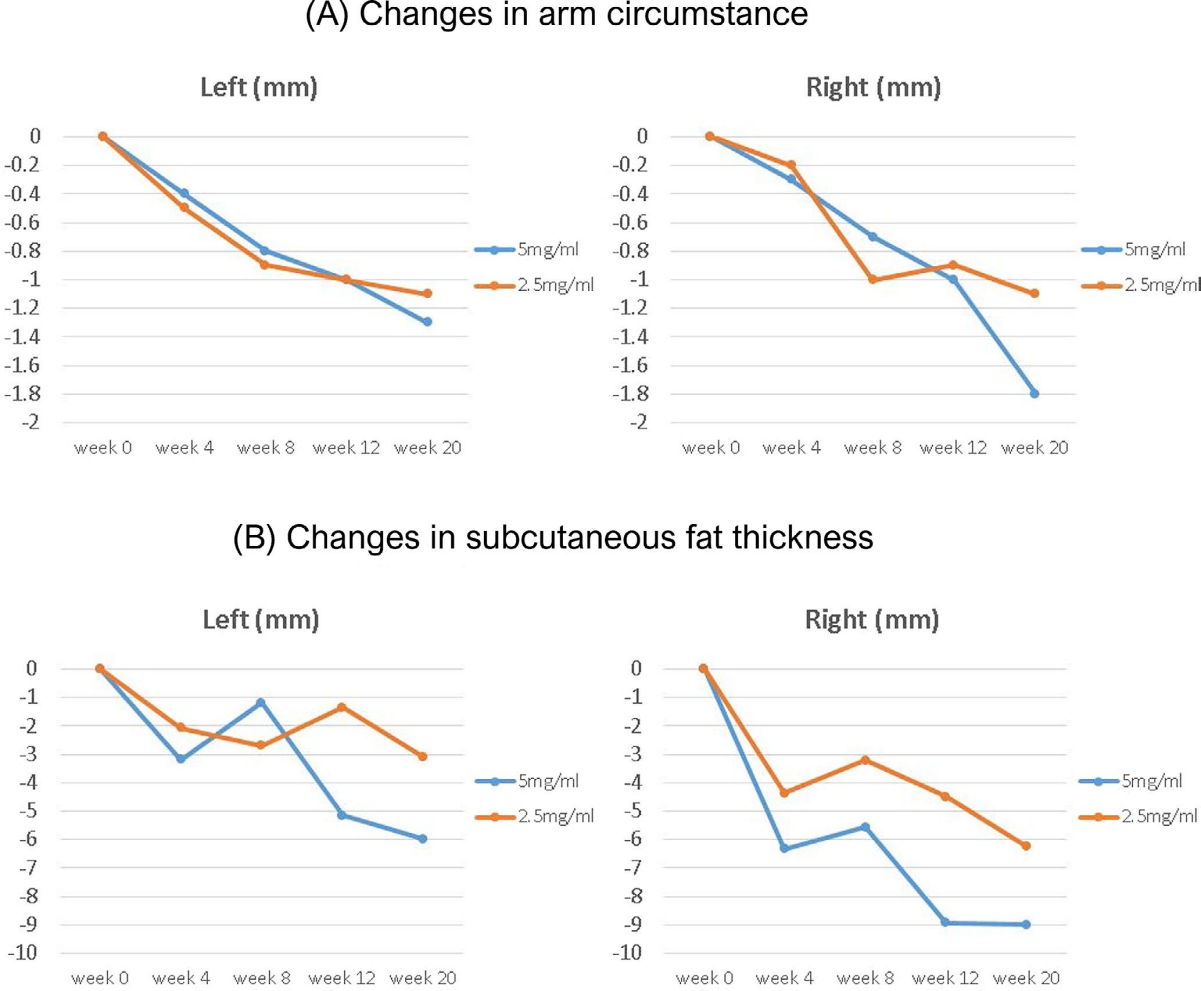
(A) Changes in arm circumference and (B) subcutaneous fat thickness were measured at baseline and at 4‐week intervals following the initiation of diluted V‐OLET injections.

**FIGURE 2:**
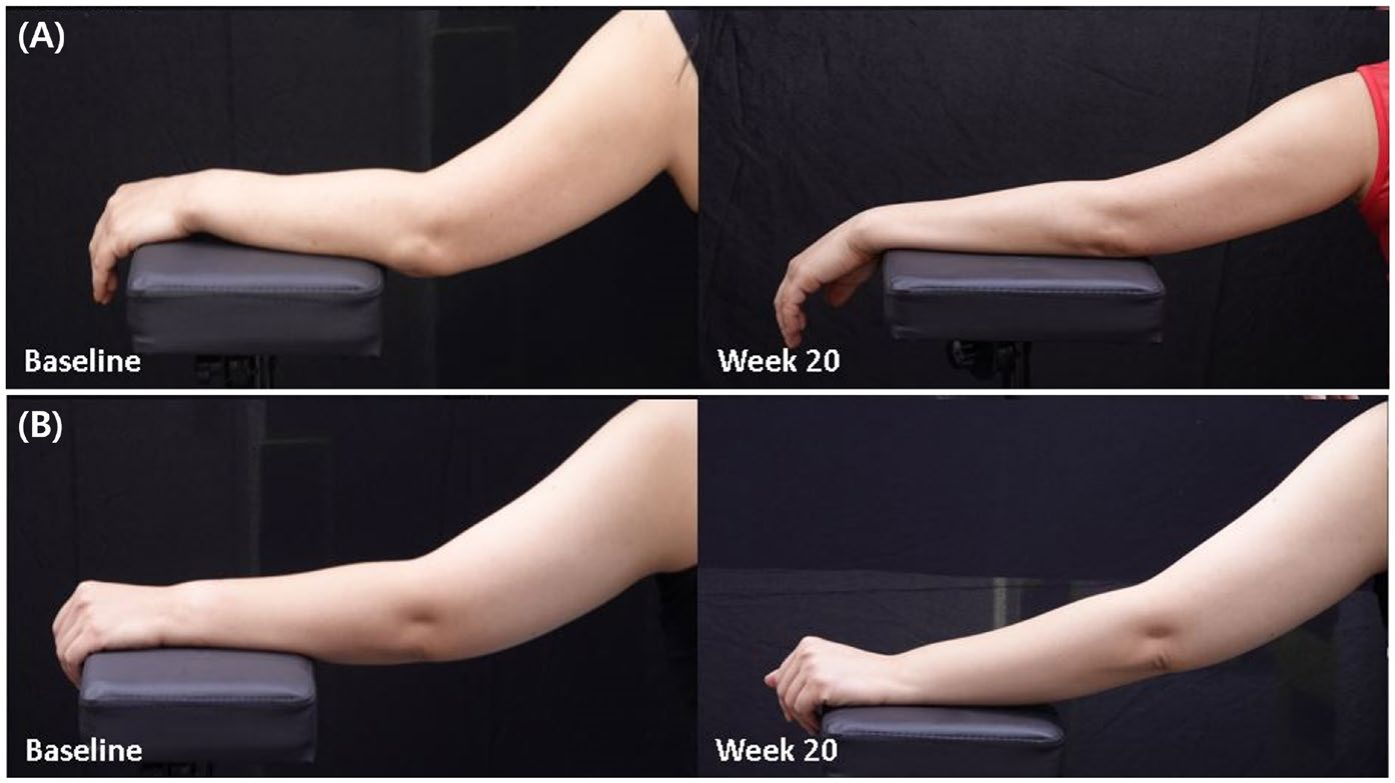
Clinical photographs of the left arm before and 20 weeks after treatment with V‐OLET injections. (A) 5 mg/mL concentration and (B) 2.5 mg/mL concentration.

**FIGURE 3:**
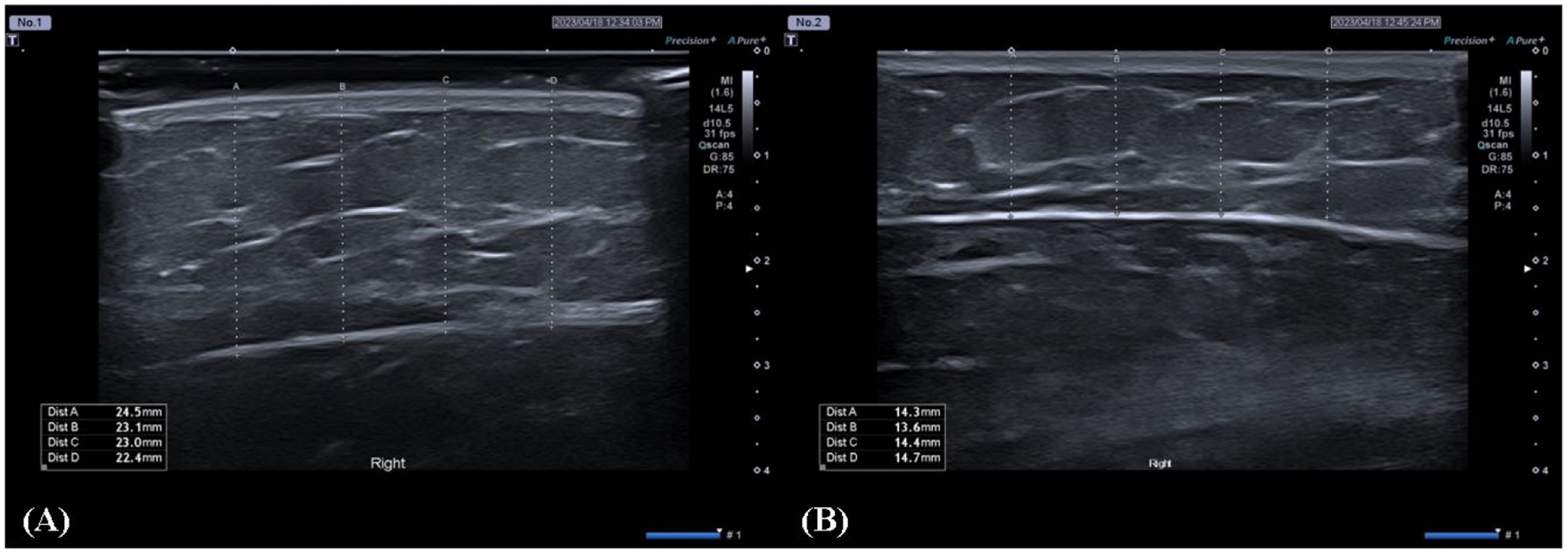
Ultrasound imaging of the arm (A) before and (B) 20 weeks after treatment with a 5 mg/mL V‐OLET injection.

Four Korean females aged 27–34 years enrolled in the study and were randomized into two groups. One patient from each group completed the follow‐up, and the results were compiled for the completed cases. After 20 weeks, the mean change in arm circumference was not significantly different between the two groups (1.1 mm in the 2.5 mg/mL injection group vs. 1.55 mm in the 5 mg/mL injection group) (Figure 1). However, the change in the mean value of subcutaneous fat thickness as determined by ultrasound was greater in the 5 mg/mL injection group than in the 2.5 mg/mL injection group (7.49 mm vs. 4.68 mm) (Figure 1). The VAS scores, reflecting pain intensity, were higher in the 5 mg/mL injection group than in the 2.5 mg/mL injection group immediately after the first dose (10 vs. 6.5), 1 week after the second dose (5 vs. 1), and 1 week after the third dose (2 vs. 0.5). 4 weeks after the last dose, the patient satisfaction level was 3 (somewhat dissatisfied) in the 5 mg/mL injection group and 1 (completely dissatisfied) in the 2.5 mg/mL injection group, with dissatisfaction noted in both groups. Table 1 provides a comparative summary of the variable values by DCA dosage. Clinical photographs and ultrasound images are shown in Figures 2 and 3.

**TABLE 1:** Comparison of variable values by DCA dosage.

Measurement	5 mg/mL DCA	2.5 mg/mL DCA
Number of participants	2	2
Age range (years)	27–34	
Mean arm circumference change (mm)^a^	1.55	1.1
Mean subcutaneous fat thickness reduction (mm)^a^	7.49	4.68
Serial change of VAS pain score^b^	10–5–2	6.5–1–0.5
Patient satisfaction	3 (Somewhat dissatisfied)	1 (Completely dissatisfied)
(4 weeks after last dose)		

^a^ Arm circumference and subcutaneous fat thickness were measured at 20 weeks post‐treatment.

^b^ Pain intensity was assessed at three time points: immediately after the first dose, 1 week after the second dose, and 1 week after the third dose.

## 4 Discussion

Deoxycholic acid (DCA) is used to reduce localized fat deposits by emulsifying phospholipids and solubilizing biological membranes, leading to fat necrosis. Previous studies have explored its use for contouring various body areas, including the buffalo hump, flanks, arms, abdomen, and inner thighs [8]. DCA is effective in removing fat from the abdomen and flanks but is less effective in removing fat from the upper arms. Therefore, this study aimed to identify the lipolytic effect of DCA in the upper arm and determine an effective and safe concentration of DCA for improving flabby arms.

At both concentrations of DCA (5 mg/mL and 2.5 mg/mL), a noticeable decrease in subcutaneous fat thickness was observed on ultrasound, with the effect persisting until week 20, i.e., 8 weeks after the last injection. However, the decrease in upper arm circumference was not significant, resulting in modest patient satisfaction. The discrepancy between the reduction in subcutaneous fat and arm circumference could be attributed to several factors. First, arm circumference is influenced not only by subcutaneous fat but also by muscle mass, skin elasticity, and other soft tissues. A reduction in subcutaneous fat may not immediately translate to a significant decrease in overall arm circumference, particularly if the muscle mass remains unchanged or if there is skin laxity. Therefore, screening patients before treatment to assess skin laxity and considering adjunctive procedures such as brachioplasty or energy‐based devices (e.g., radiofrequency, high‐intensity focused ultrasound [HIFU], and lasers) may be essential to achieve optimal contouring outcomes [9, 10]. Second, our study population primarily comprised individuals with a relatively low BMI; thus, they initially had a relatively small amount of subcutaneous fat. Therefore, even a measurable reduction in fat thickness might not lead to a noticeable change in arm circumference. Future studies could include participants with a broader range of BMIs to assess whether higher initial fat volumes yield more noticeable reductions in arm circumference.

Given the limitations of DCA monotherapy, combination therapy may enhance clinical outcomes. Low‐level laser therapy decreases the circumference of the upper arm by stimulating the production of cyclic adenosine monophosphate (cAMP) by activating cytochrome C oxidase. This activation leads to the breakdown of cellular lipids within adipocytes, creating temporary pores in their cell membrane and causing cell collapse [11]. Additionally, phosphatidylcholine, a glycerophospholipid, stimulates lipases, resulting in triglyceride breakdown into fatty acids and glycerol. When combined with DCA, phosphatidylcholine has demonstrated improved efficacy in reducing localized subcutaneous fat accumulation [12]. These combination approaches may enhance the overall effectiveness of nonsurgical upper arm contouring.

A common side effect of DCA is pain at the injection site. While the reduction in subcutaneous fat thickness was greater with the 5 mg/mL concentration compared to the 2.5 mg/mL concentration, the higher concentration was also associated with increased pain levels. This was consistent with data from two participants who were lost to follow‐up. Given the pain intensity, the 2.5 mg/mL concentration of DCA may be preferable in some cases. To improve patient comfort, several pain management strategies can be utilized. Pre‐treatment NSAIDs (e.g., ibuprofen) help reduce pain and inflammation, while topical lidocaine applied 15–30 min before injection provides localized numbness. Injectable lidocaine with epinephrine can further minimize pain and bruising. Cold application before and after treatment reduces swelling, and proper patient positioning and injection technique help minimize tissue trauma. Implementing these measures can enhance procedural tolerance and reduce injection‐related discomfort [13].

One of the key limitations of our study is the absence of a control group. A placebo or untreated control group would have provided a more definitive assessment of DCA's efficacy by ruling out confounding variables such as natural fluctuations in weight or localized fat distribution. Furthermore, long‐term follow‐up is needed to determine whether DCA‐induced fat reduction leads to progressive changes in skin texture and firmness. Future studies could also assess metabolic markers related to adipocyte apoptosis and inflammation to better understand the biological mechanisms underlying treatment efficacy.

In conclusion, diluted DCA injections demonstrated partial effectiveness in reducing subcutaneous fat in the upper arms, with no major adverse effects. However, the limited reduction in arm circumference suggests that DCA alone may not be sufficient for comprehensive upper arm contouring. While the 5 mg/mL concentration showed greater fat reduction than the 2.5 mg/mL concentration, it was also associated with increased pain, highlighting a trade‐off between efficacy and tolerability. Additionally, the cost of higher doses may be a limiting factor in clinical application. Given these findings, clinicians should consider patient tolerance, treatment cost, and expected outcomes when selecting DCA concentrations. Combining DCA injections with other noninvasive fat reduction techniques, such as radiofrequency, HIFU, or cryolipolysis, may enhance overall contouring effects. Future controlled trials with larger, more diverse populations and extended follow‐up periods are needed to further evaluate the long‐term efficacy and safety of DCA for upper arm contouring.

## Author Contributions

**Ji Yeon Hong:** conception and design of the study, drafting the manuscript, and critical revision of the manuscript for important intellectual content. **Yoon Hwan Lee:** data collection, analysis, and interpretation of results, as well as contributions to drafting sections of the manuscript. **Seung Hoon Yeoum:** data acquisition and coordination of the clinical aspects of the study, assisting in manuscript preparation and revision. **Choon Shik Youn:** supervision of clinical trials, providing clinical insights, and assisting with interpretation of data and manuscript revisions. **Kui Young Park:** oversight of the entire project, final approval of the manuscript, and contributions to the conception and design of the study, as well as critical revision of the manuscript.

## Ethics Statement

This study was conducted in accordance with the principles of the Declaration of Helsinki. The institutional review board at Chung‐Ang University Hospital approved all study protocols, informed consent forms, and relevant supporting data (IRB‐2306‐010‐558). Written informed consent was obtained from all participants prior to their inclusion in the study. All procedures were performed in compliance with relevant guidelines and regulations. No animals were used in this research.

## Conflicts of Interest

Dr. Kui Young Park and Dr. Choon Shik Youn are consultants and speakers for Daewoong Pharmaceuticals Inc., and the remaining authors declare no conflicts of interest.

## Data Availability

The data that support the findings of this study are available on request from the corresponding author. The data are not publicly available due to privacy or ethical restrictions.

## References

[1] N. Nagrath and R. Winters , “Brachioplasty,” in StatPearls (StatPearls Publishing, 2025), https://www.ncbi.nlm.nih.gov/books/NBK585115/?utm_source=chatgpt.com. 36256762

[2] J. D. Carruthers , S. Humphrey , and J. K. Rivers , “Cryolipolysis for Reduction of Arm Fat: Safety and Efficacy of a Prototype Coolcup Applicator With Flat Contour,” Dermatologic Surgery 43, no. 7 (2017): 940–949. 28595246 10.1097/DSS.0000000000001134 PMC5491235

[3] S. J. Lee , H. W. Jang , H. Kim , D. H. Suh , and H. J. Ryu , “Non‐Invasive Cryolipolysis to Reduce Subcutaneous Fat in the Arms,” Journal of Cosmetic and Laser Therapy 18, no. 3 (2016): 126–129. 26735803 10.3109/14764172.2015.1114644

[4] J. S. Tan , C. C. Lin , J. S. Cheng , and G. S. Chen , “High‐Intensity Focused Ultrasound Ablation Combined With Electrical Passive Exercise for Fast Removal of Body Fat,” Plastic and Reconstructive Surgery 145, no. 6 (2020): 1427–1438. 32195859 10.1097/PRS.0000000000006826

[5] E. D. Deeks , “Deoxycholic Acid: A Review in Submental Fat Contouring,” American Journal of Clinical Dermatology 17, no. 6 (2016): 701–707. 27785706 10.1007/s40257-016-0231-3

[6] S. M. Jegasothy , “Deoxycholic Acid Injections for Bra‐Line Lipolysis,” Dermatologic Surgery 44, no. 5 (2018): 757–760. 29016540 10.1097/DSS.0000000000001311 PMC5943074

[7] J. M. Sykes , A. Allak , and B. Klink , “Future Applications of Deoxycholic Acid in Body Contouring,” Journal of Drugs in Dermatology 16, no. 1 (2017): 43–46. 28095531

[8] R. Amore , D. Amuso , V. Leonardi , et al., “Evaluation of Safe and Effectiveness of an Injectable Solution Acid Deoxycholic Based for Reduction of Localized Adiposities,” Plastic and Reconstructive Surgery. Global Open 6, no. 6 (2018): e1794. 30276043 10.1097/GOX.0000000000001794 PMC6157955

[9] E. A. Appelt , J. E. Janis , and R. J. Rohrich , “An Algorithmic Approach to Upper Arm Contouring,” Plastic and Reconstructive Surgery 118, no. 1 (2006): 237–246. 16816702 10.1097/01.prs.0000231933.05534.95

[10] B. Teimourian and S. Malekzadeh , “Rejuvenation of the Upper Arm,” Plastic and Reconstructive Surgery 102, no. 2 (1998): 545–553. 9703097 10.1097/00006534-199808000-00041

[11] M. Nestor , B. Zarraga , and H. Park , “Effect of 635nm Low‐Level Laser Therapy on Upper Arm Circumference Reduction a Double‐Blind, Randomized, Sham‐Controlled Trial,” Journal of Clinical and Aesthetic Dermatology 5, no. 2 (2012): 42–48. PMC3315881 22468172

[12] M. K. Thomas , J. A. D'Silva , and A. J. Borole , “Injection Lipolysis: A Systematic Review of Literature and Our Experience With a Combination of Phosphatidylcholine and Deoxycholate Over a Period of 14 Years in 1269 Patients of Indian and South East Asian Origin,” Journal of Cutaneous and Aesthetic Surgery 11, no. 4 (2018): 222–228, 10.4103/jcas.jcas. 30886477 PMC6371720

[13] S. Fagien , P. Mc Chesney , M. Subramanian , and D. H. Jones , “Prevention and Management of Injection‐Related Adverse Effects in Facial Aesthetics: Considerations for ATX‐101 (Deoxycholic Acid Injection) Treatment,” Dermatologic Surgery 42 (2016): S300–S304, 10.1097/dss.0000000000000898. 27787270

